# Application of maple on computing strong fuzzy chromatic polynomial of fuzzy graphs

**DOI:** 10.1186/s13104-022-06242-6

**Published:** 2022-11-08

**Authors:** Mamo Abebe Ashebo, Laxmi Rathour, V. N. SrinivasaRao Repalle

**Affiliations:** 1 Department of Mathematics, Wollega University, Nekemte, Ethiopia; 2 Ward Number-16, Bhagatbandh, Anuppur, 484224 Madhya Pradesh India

**Keywords:** Strong fuzzy chromatic polynomial, Strong fuzzy chromatic number, Fuzzy graph, Maple, 05C72, 05C15, 05C31

## Abstract

**Objective:**

In the field of graph theory, maple is a technical computation form that is used for solving problems. In this article, we apply maple to find the strong fuzzy chromatic polynomial of fuzzy graphs and related. Moreover, we apply maple to obtain strong fuzzy chromatic numbers of fuzzy graphs using their strong fuzzy chromatic polynomials.

**Results:**

The strong fuzzy chromatic polynomials for fuzzy graphs, strong fuzzy graphs and complete fuzzy graphs are determined using maple. Furthermore, the strong fuzzy chromatic numbers for the fuzzy graphs are obtained.

## Introduction

A graph is a convenient way of representing information involving relationship between objects. The objects are represented by vertices and relations by edges. To handle the uncertainty and incompleteness in the description of the objects or in its relationships or in both, Zadeh [1] introduced a fuzzy set theory. Rosenfeld [2] developed the theory of fuzzy graph based on Zadeh’s fuzzy set and fuzzy relations. After that, several scholars introduced many concepts in the fuzzy graph theory [3–12]. Mathematical tools play a vital role in solving real-world problems. Fuzzy coloring is very useful tools for solving many problems including traffic light problems [13–16]. Numerous researchers published their work on the fuzzy coloring and related concepts [17–19]. Based on strong arcs, the concept of strong coloring of fuzzy graphs has been introduced by Kishore and Sunitha [20]. In recent times, the notion of fuzzy chromatic polynomial and its properties [21, 22] and the notion of strong fuzzy chromatic polynomial of fuzzy graphs [23] have been studied by scholars.

Maple’s Graph Theory package was developed by a group of graduate students and faculty at Simon Fraser University under the direction of Michael Monagan starting 2004. The design of the package was first presented at the 2005 Maple conference in Waterloo in the summer of 2005. New commands and improvements, in particular to facilities for drawing graphs, were presented at the 2006 Maple conference [24, 25]. The first version of the package was released in Maple 11 in 2007 as the Graph Theory package. The package supports simple undirected graphs and simple directed graphs, both of which may be weighted.

A few operations of graphs like fuzzy graph, wiener index of graph, cluster and corona operations of graph, total graph, semi-total line and edge join of graphs have been valuable in graph theory and chemical graph theory to consider the properties of boiling point, heat of evaporation, surface tension, vapor pressure, total electron energy of polymers, partition coefficients, ultrasonic sound velocity and internal energy. Monte Carlo simulation technique is used in survival signature to analyze the fuzzy reliability of systems having complexity [26–31]. The degree sequence of a graph and algebraic structure of different graphs operations were determined and its result is to the join and corona products of any number of graphs.

For the notations not declared in this manuscript, to understand well we recommend the readers to refer [32–36]

In this article, we apply maple to find the strong fuzzy chromatic polynomials of fuzzy graphs including strong and complete fuzzy graphs. Moreover, we obtain the strong fuzzy chromatic number for fuzzy graphs based on their strong fuzzy chromatic polynomials.

## Main text

### Method

#### Definition 1

[23] Let $G$ be a fuzzy graph with a positive integer $k$, the number of distinct $k$-strong colorings of $G$ is called strong fuzzy chromatic polynomial (SFCP) of $G$.

It is denoted by ${P}_{s}^{f}\left(G,k\right)$.

For a fuzzy graph $G$, ${P}_{s}^{f}\left(G,k\right)=P({G}^{*},k)$ iff all the arcs in $G$ are strong or ${P}_{s}^{f}\left(G,k\right)=P({H}^{*},k)$ if $G$ has at least one $\delta$-arc and $H=G-\left\{\delta -arcs\right\}.$ Here, we observed that the strong fuzzy chromatic polynomials of fuzzy graphs $G$ are determined in terms of the chromatic polynomial of crisp graphs ${G}^{*}$ or ${H}^{*}$.

#### Theorem 1

[23] Let $G$ be a strong fuzzy graph and ${G}^{*}$ be its underlying crisp graph. Then ${P}_{s}^{f}\left(G,k\right)=P\left({G}^{*},k\right).$

#### Theorem 2

[23] Let $G$ be a complete fuzzy graph with* n* vertices*.* Then ${P}_{s}^{f}\left(G,k\right)=P\left({K}_{n}, k\right),$ where ${K}_{n}$ is a complete crisp graph with* n* vertices.

### Strong fuzzy chromatic polynomial by maple

In this article, for computing the chromatic polynomial of a crisp graph, we use Maple 18 which was released in 2014. In the Graph Theory package, the chromatic polynomial of a crisp graph is determined by using a Maple command, called “*P:* = *ChromaticPolynomial(G,k)*”.

#### Example 1

**Fig. 1:**
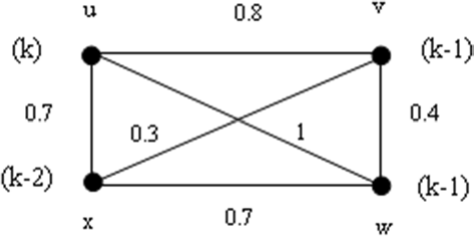
A fuzzy graph $$G$$

Find the strong fuzzy chromatic polynomial the fuzzy graphs in Fig. 1 by Maple.

**Solution:** For the fuzzy graph $G$ in Fig. 1, ${P}_{s}^{f}\left(G,k\right)=P({H}^{*},k)$, where $H=G-\left\{\left(v,w\right), (v,x)\right\}$. Thus, to compute, $P({H}^{*},k)$, we will give it by the following steps of Maple commands in Maple 18.

 > *with(GraphTheory):*

 > $${H}^{*}$$: = *Graph([u,v,w,x]*,

*{{u,v},{u,w},{u,x},{x,w}}):*

*> DrawGraph(* ${H}^{*}$ *, style = circle):*

*> P: = ChromaticPolynomial(* $${H}^{*}$$ *, k)*

1 $$P: = k{\left( {k - 1} \right)^2}(k - 2)$$

Therefore, $${P}_{s}^{f}\left(G,k\right)=k{\left(k-1\right)}^{2}(k-2)$$.

#### Example 2

**Fig. 2:**
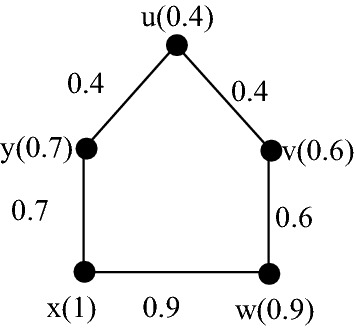
A strong fuzzy graph $G$

**Fig. 3:**
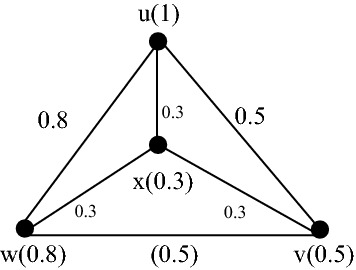
A complete fuzzy graph $G$

Determine the strong fuzzy chromatic polynomials of the fuzzy graphs in Fig. 2 and 3 using Maple.

**Solution**: Since the fuzzy graph in Fig. 2 is strong, by Theorem 1${P}_{s}^{f}\left(G,k\right)=P\left({G}^{*},k\right).$ Therefore, to compute, $P({G}^{*},k)$, we will give it by the following steps of Maple commands in Maple 18.

 > *with(GraphTheory):*

 > $${G}^{*}$$: = *Graph([u,v,w,x,y], {{u,v},{v,w},{w,x},{x,y},{y,u}}):*

 > *DrawGraph(*${G}^{*}$*, style* = *circle):*

 > *P:* = *ChromaticPolynomial(*$${G}^{*}$$*,k)*

2 $$P:={\left(k-1\right)}^{5}-k+1$$

Hence*, *$${{P}_{s}^{f}\left(G,k\right)=\left(k-1\right)}^{5}-k+1$$*.*

Similarly, since the fuzzy graph in Fig. 3 is complete, by Theorem 2${P}_{s}^{f}\left(G,k\right)=P\left({K}_{4},k\right).$ Therefore, to compute, $P({K}_{4},k)$, we will give it by the following steps of Maple commands in Maple 18.

 > *with(GraphTheory):*

 > *with(SpecialGraphs):*

 > *G:* = *CompleteGraph(4):*

 > *DrawGraph(G, style* = *planar):*

 > *P:* = *ChromaticPolynomial(G,k)*

3 $$P:=k\left(k-1\right)\left(k-2\right)(k-3)$$

Thus*,*$${P}_{s}^{f}\left(G,k\right)=k\left(k-1\right)\left(k-2\right)(k-3)$$.

### Strong fuzzy chromatic number by maple

Besides counting of the number of strong colorings on fuzzy graphs, the strong fuzzy chromatic polynomials can be used to obtain strong fuzzy chromatic number of a fuzzy graph.

The following definition finds the strong fuzzy chromatic number, ${\chi }_{s}^{f}(G)$ from the strong fuzzy chromatic polynomial ${P}_{s}^{f}\left(G,k\right).$

#### Definition 2

[23] Let $G$ be a fuzzy graph*.* The number $k$ is called the strong fuzzy chromatic number of $G$ if there exist the smallest positive integer $k$ such that ${P}_{s}^{f}\left(G,k\right)\ne 0.$ In this case, $k={\chi }_{s}^{f}(G)$.

Maple tools can be used for finding the strong fuzzy chromatic number of a fuzzy graph, ${\chi }_{s}^{f}\left(G\right)$. To find ${\chi }_{s}^{f}\left(G\right)$ we use a Maple command, called “*eval(P, k)*”, which means evaluate the chromatic polynomial $P$ at positive integer $k$. Finally, by using Definition 2 we obtain ${\chi }_{s}^{f}\left(G\right)$. The situation is illustrated by numerical examples.

#### Example 2

Consider the fuzzy graph in Fig. 1. Find ${\chi }_{s}^{f}\left(G\right)$ by Maple.

**Solution**: First, consider the fuzzy graph $G$ in Fig. 1., the Maple commands to find ${\chi }_{s}^{f}\left(G\right)$ are

 > *with(GraphTheory):*

 > $${H}^{*}$$: = *Graph([u,v,w,x], {{u,v},{u,w},{u,x},{x,w}}):*

*> DrawGraph(* ${H}^{*}$ *, style = circle):*

*> P: = ChromaticPolynomial(* $${H}^{*}$$ *,k)*

4 $$P:=k{\left(k-1\right)}^{2}(k-2)$$

 > *eval(P, k* = *2)*

5 $$0$$

 > *eval(P, k* = *3)*

6 $$12$$

Here, the smallest positive integer $k$ so that ${P}_{s}^{f}\left(G,k\right)\ne 0$ is 3. Therefore, by Definition 2${\chi }_{s}^{f}\left(G\right)=3.$

#### Example 3

Consider the fuzzy graphs in Fig. 2 and Fig. 3. Find ${\chi }_{s}^{f}\left(G\right)$ by Maple.

First, consider the fuzzy graph $G$ in Fig. 2. The Maple commands to find ${\chi }_{s}^{f}\left(G\right)$ are as follows.

 > *with(GraphTheory):*

 > $${G}^{*}$$: = *Graph([u,v,w,x,y], {{u,v},{v,w},{w,x},{x,y},{y,u}}):*

 > *DrawGraph(*${G}^{*}$*, style* = *circle):*

 > *P:* = *ChromaticPolynomial(*$${G}^{*}$$*,k)*

7 $$P:={\left(k-1\right)}^{5}-k+1$$

 > *eval(P, k* = *2)*

8 $$0$$

 > *eval(P, k* = *3)*

9 $$30$$

Here, the smallest positive integer $k$ so that ${P}_{s}^{f}\left(G,k\right)\ne 0$ is 3. Therefore, by Definition 2${\chi }_{s}^{f}\left(G\right)=3.$

Similarly, consider the fuzzy graph $G$ in Fig. 3, the Maple commands to find ${\chi }_{s}^{f}\left(G\right)$ is as follows.

 > *with(GraphTheory):*

 > *with(SpecialGraphs):*

 > *G:* = *CompleteGraph(4):*

 > *DrawGraph(G, style* = *planar):*

 > *P:* = *ChromaticPolynomial(G,k)*

10 $$P:=k\left(k-1\right)\left(k-2\right)(k-3)$$

 > *eval(P, k* = *3)*

11 $$0$$

 > *eval(P, k* = *4)*

12 $$24$$

Here, the smallest positive integer $k$ so that ${P}_{s}^{f}\left(G,k\right)\ne 0$ is 4. Therefore, by Definition 2${\chi }_{s}^{f}\left(G\right)=4.$

### Conclusion

The findings of the present study showed that the maple software can be applied to obtain the strong fuzzy chromatic polynomials of fuzzy graphs and to find their strong fuzzy chromatic numbers when the existing methods were not applicable. In the future work, we will apply Maple tool to compute the chromatic polynomial of the Intuitionistic Fuzzy Graphs.

### Limitations

This study focuses on how to use the maple software on some fuzzy graphs to find out the strong fuzzy chromatic polynomial, and strong fuzzy chromatic number. The results obtained by maple are exactly the same as the results obtained by the existing methods. The maple software can be used and more meaningful when the number of vertices and edges in a fuzzy graph is increased.

## Data Availability

The authors declare that the data supporting the findings are included in the paper.
